# The Use of Mercurol in Urethritis

**Published:** 1900-11

**Authors:** 


### THE USE OF MERCUROL IN URETHRITIS.

RAMON GUITERAS in *The Lancet* (London, England, September 22, 1900,) states that he has thoroughly tried mercurol in his clinic, and from his experience has drawn certain conclusions which he presents in his paper. After describing the chemical nature of mercurol, he states that he found the weaker solutions had little effect and the stronger solutions were at first irritating. He finally concluded that the average strength best borne by the patient is ten grains to the ounce, or approximately 2 per cent. After having reached this conclusion he had the histories of 100 cases recorded, in 33 of which an examination for the gonococcus was made, revealing its presence in 30 cases. In the remaining 67 cases a clinical diagnosis was depended upon, since the writer considers the experienced eye competent to recognise the disease. In one extremely interesting case no gonococcus could be found in the urethral discharge, although gonococci were present in that of some venereal ulcers on the glans.

In these cases a 2 per cent. solution of mercurol was ordered which the patients were directed to inject three times a day, after micturition; the injection to be held within the urethra for five minutes at each operation. The clinical reports of the cases show that frequently in two days after beginning the use of mercurol, gonococci could no longer be found in the discharge.

The author discusses at some length the value of the term "practically cured," and sums up his argument by saying that to draw conclusions of value we should consider only cases that have been under treatment for three or more weeks, omitting those making but a few visits. On this basis he eliminates all but 65 cases from his report and tabulates these as follows:

Ten cases were cured in two weeks, or 15 per cent.; fifteen cases were cured in six weeks, or 23 per cent.; twenty cases were practically cured, as there was no discharge, though there were some shreds in the urine at the end of from four to eight weeks, 30 per cent.

One of the most valuable observations that the writer has made is the fact that only two cases suffered from complications, one having developed gonorrheal rheumatism and the other epididymitis. He states that this fact in itself would tend to argue much in favor of the use of mercurol, for where is there any other solution or mixture which does not show a greater percentage of complications? When we consider that many writers claim that epididymitis occurs in 20 per cent. of all cases of urethritis, the rate of 1 per cent. reported in this series of cases argues much in favor of mercurol as a harmless, yet efficient injection.

Another interesting feature is that in only one of the 100 cases was there any marked posterior urethritis. Therefore, it would seem that mercurol quickly destroyed the gonococcus, lessens the severity of the inflammation, and tends to prevent the development of complications. From a comparative study of the different methods of treating gonorrhea, the author concludes that treatment with mercurol is an advance beyond the older methods with balsamics and astringent injections.

